# Cross-border collaboration for neglected tropical disease efforts—Lessons learned from onchocerciasis control and elimination in the Mano River Union (West Africa)

**DOI:** 10.1186/s12992-016-0185-5

**Published:** 2016-08-22

**Authors:** Kenneth Gustavsen, Yao Sodahlon, Simon Bush

**Affiliations:** 1 Merck & Co., Inc., 2000 Galloping Hill Road, Kenilworth, NJ 07033 USA; 2 Mectizan Donation Program, 325 Swanton Way, Decatur, GA 30030 USA; 3 Sightsavers, Airport, PO Box 18190, Accra, Ghana

**Keywords:** Onchocerciasis, River blindness, Neglected tropical diseases, NTDs, Africa, Health, Partnership, WHO, Collaboration, Cross-border

## Abstract

Diseases don’t respect borders, so efforts to control and eliminate diseases must also be flexible and adaptable enough to effectively reach the populations that live in the areas around national frontiers. Onchocerciasis, commonly known as river blindness is a tropical disease that has historically affected millions of people in 35 countries in Africa and Latin America. In Africa, programs and partnerships to address river blindness through mass drug administration have been active for more than 25 years. While in many cases the disease is found in isolated foci that fall entirely within national boundaries, the geographic scope of many affected areas crosses country borders. National river blindness programs are the responsibility of each nation’s Ministry of Health, so in cross-border situations there is a need for effective country-country collaboration. Cross-border collaboration for onchocerciasis control efforts in the countries of the Mano River Basin illustrates the positive impact of a creative model, and offers lessons for expanded application for onchocerciasis elimination as well as other neglected tropical disease (NTD) control and elimination programs.

## Background on river blindness

Onchocerciasis, commonly known as river blindness, is a neglected tropical disease (NTD) that is currently endemic in 35 countries in Africa, Latin America and the Middle East [1]. The disease is caused by a parasite, *Onchocerca volvulus*, which is transmitted by the bite of infected black flies that breed in fast-flowing rivers. Infection in humans can lead to skin depigmentation and loss of elasticity, painful nodules under the skin where the adult parasites are lodged, and vision loss and eventual blindness when the parasites migrate to the eyes [2]. Initial efforts to control onchocerciasis in Africa were through the Onchocerciasis Control Program (OCP) of the World Health Organization (WHO), an 11 country effort in West Africa to control the disease initially through vector control that ran from 1975 to 2002 [3]. While vector control had positive impact on controlling onchocerciasis, since 1987, the preferred control strategy for onchocerciasis is annual or semi-annual treatment with ivermectin, which kills the juvenile parasites. With sufficient treatment coverage at the community level over time, the cycle of transmission can eventually be broken and the disease eliminated [1]. A second WHO regional program in Africa, the African Program for Onchocerciasis Control (APOC), ran from 1995 to 2015 to support the control efforts in 19 countries outside of the OCP area (although there was some support offered for ex-OCP countries) [4]. Onchocerciasis is one of the 10 NTDs targeted for elimination through the WHO’s Roadmap on NTDs [5]. Achieving elimination requires sustained annual treatment coverage rates of >80 % at the community level for a period of 15–20 years [1, 6].

### Issues associated with cross-border coordination

Cross-border collaboration between sovereign nations is a complex undertaking for any issue. For a disease control and elimination program such as one for onchocerciasis, there is a range of issues to be considered, including mapping of endemic areas including degrees of endemicity, and reaching contiguous affected areas regardless of national borders to ensure accurate and complete treatment coverage. Treatments are typically coordinated according to seasonal and other considerations. Cross-border synchronization of Mass Drug Administration (MDA) can allow for more effective impact within communities given the lifecycle of the parasite *Onchocerca volvulus* and the associated vector dynamics, and the relatively free movement of people between neighboring countries. Synchronization of MDA can ensure that the lifecycle of the parasite is broken in the shared focus in a coordinated and widespread manner, thereby preventing reinfection from a neighboring area where the lifecycle was not similarly impacted. Similarly, as some countries consider a move from annual to semi-annual treatment frequency, cross-border alignment is necessary to ensure complementary impact on the parasite population. As programs near the threshold for stopping treatment and determining if transmission has been halted, all adjacent, contiguous areas must be at the same endemicity point to prevent recrudescence [1].

Recognizing the importance of cross-border collaboration for the success of the control/elimination of onchocerciasis in Africa, the WHO’s Africa Regional Office (WHO AFRO) Regional Committee approved in 2007 the resolution AFR/RC57/R3 calling on member states to intensify cross-border activities to strengthen surveillance and avoid spillage of infection to freed zones [7]. While the resolution calls for improved cross-border collaboration, it does not provide any guidance for how countries are to engage in that necessary but challenging coordination process.

Given the widespread nature of onchocerciasis across dozens of countries in Africa, there are many cross-border challenges and opportunities involving onchocerciasis MDA. The evolution of efforts to address onchocerciasis within the adjacent geography of three countries within the Mano River Union: Sierra Leone, Liberia and Guinea (Conakry), provides valuable lessons of how cross-border collaboration can evolve to enable effective MDA. Those lessons can be further refined to develop a framework that can be applied more generally, currently unexamined through this lens, providing an opportunity for cross-border collaboration to ensure that health gains on one side of the border are secured through an appropriate cross-border integrated strategy.

### Case Study: Sierra Leone, Liberia and Guinea (Conakry)/Mano River Union

The Mano River Union (MRU) is an intergovernmental body originally established between Liberia and Sierra Leone in 1973, expanded to include Guinea (1980) and later extended to Cote d’Ivoire (2008). Organized in relation to the members’ shared connection to the Mano River, the MRU “aims to strengthen the capacity of Member States to integrate their economies and coordinate development programmes in the areas of peace building, as a prerequisite to any development, trade promotion, development of industry, energy, agriculture, natural resources, transport and telecommunications, monetary and financial affairs in short, all aspects of economic and social life of the Member States” [8].

As a result of a suggestion by MRU’s Secretary General to the Sightsavers’ Regional Director in early 2005 to utilize the structures of the MRU to support work on river blindness in border areas, the mandate of the MRU was extended in 2006 to include health. MRU member states are active participants in the MRU river blindness meetings, “which convene the ministries of health and technical partners in Guinea, Cote d’Ivoire, Liberia, and Sierra Leone to discuss and resolve cross-border NTD issues. Coordination of NTD treatment among countries with shared mobile populations enables each country to support the disruption of disease transmission on a regional level” [9].

A need had been identified to increase MDA coverage against river blindness in the border areas of the MRU’s member states—in particular between Liberia and Sierra Leone following the post–conflict environment of both countries as expressed in 2006 in a declaration on Onchocerciasis Control in Africa signed by African ministers of health in Yaoundé [10]. The direct request was for the MRU to increase its scope from political and economic integration to that of health integration and coordination. Sierra Leone and Guinea were previously members of the OCP umbrella of onchocerciasis control efforts in West Africa, providing a foundation for this new effort to build on [3]. Following a period of advocacy and negotiation the first meeting, attended by Liberia, Sierra Leone and Guinea (Conakry), was held in late 2005. By 2008 the meetings had moved beyond information sharing and protocol and had started to make the following recommendations:

- Onchocerciasis control teams in the three countries should visit each other across the borders in a bid to share knowledge and experience.
- Sierra Leone should conduct evaluations along the border villages with Guinea so as to obtain the exact picture of the onchocerciasis prevalence in those villages.
- Members of the tripartite meeting should to come together and mobilize resources for the implementations of the programmes.
- Guinea shall give a technical back stop to Sierra Leone and Liberia in the bid to achieve a higher coverage.
- Member countries should institute strong Monitoring and Evaluation teams that will ensure that all endemic villages are treated [11].

A meeting in 2012 went further and clearly identified the following general challenges facing all countries, particularly in the border areas:

- Lack of coordination of NTDs interventions
- Insufficient Community Directed Distributor (CDD) motivation and training
- Inadequate data management
- Insufficient monitoring and evaluation at the MRU border areas
- Inadequate logistical support

In response to the challenges raised in the 2012 meeting a technical coordination committee was established that included a representative from the NTD coordinator from the ministry of health in each country [Guinea (Conakry), Liberia, Sierra Leone and Cote d’Ivoire], a representative from the MRU Secretariat, and one non-governmental development organization (NGDO) partner (Sightsavers). This technical committee would be the link from the annual meeting to the field-level operations. Following the initial support by Sightsavers, follow on participation and support has come from a number of partners including non-governmental organizations such as Helen Keller International and academic partners such as the Filarial Programmes Support Unit (FPSU) at the University of Liverpool Tropical Medicine.^1^

With trust established and the protocols developed for river blindness, the terms of reference for the annual meeting was changed to a mandate for neglected tropical diseases and the collaboration on cross-border efforts to control five neglected tropical diseases (NTDs): lymphatic filariasis; schistosomiasis, trachoma, onchocerciasis and soil-transmitted helminthiasis [9].

Over time, the meetings have moved from information sharing to providing technical advice to national programmes from each of the countries represented. Although member states are taking increased ownership of their programs, much of the funding for the meetings still comes from program partners, such as implementing non-governmental organizations and donors. Programs have been strengthened during this 8-year period, with the cross border work conducted as part of the exchange of information, ideas and technical skills, with the MRU playing a coordination role [12].

Despite the significant progress, the process has not been simple, with every step compounded by issues of protocol and language. It was only in 2013, for example, that it was a requirement for all documentation to be in both English and French. The 2013 recommendations show how far the collaboration has advanced since the first meeting in 2005 [9]:

- All communications should be bilingual
- Countries should use the MRU secretariat for higher level advocacy on cross border control with political bodies/individuals
- Medical providers working on borders should submit information on the border populations for planning local border health authorities before end of 2013
- Countries should order sufficient MDA drugs for border villages, including potential MDA-migrants; reconcile orders quantities with MDA denominator local border health authorities before next drug order
- Countries should ensure that the MRU and cross border meetings are included in budgets and annual work plans
- MRU NTD committee should conduct a midterm review of recommendations
- Countries should recognize that MDA synchronization is optimal, despite the challenges of depending on donor support
- Countries should make provisions for district officers along the border to have quarterly meetings with cross-border neighbours
- Countries should strengthen national NTD task forces and ensure that they meet at least annually
- Country NTD programs should expand the information shared with MRU to include socio-economic impact of NTDs.

The initial modest meetings and efforts at coordination between countries on NTDs helped to promote the role of the broader MRU itself, which by 2005-6 was a mere shadow of the dream of integration. In 2008 plans to revitalize the Union were put in place that included a stress on health and development and a unified approach to, for example, national immunization programmes. Following the outbreak of Ebola virus disease (EVD) in Sierra Leone, Liberia and Guinea (Conakry) the MRU was able to coordinate cross-border work to help contain the disease and has played an important part in high level advocacy and reducing the administrative processes to allow for cross border work to be coordinated [12]. Indeed, the Ebola crisis has illustrated the need for enhanced community engagement and education to ensure MDA is conducted in a way that reflects the new sensitivities to public health challenges by the general public, especially in areas where people regularly cross national borders as part of their daily routine. As MDA resumes in the MRU countries for river blindness and the other neglected tropical diseases following the outbreak of Ebola virus disease in three of the four countries of the MRU, the need for regional collaboration remains vital. For example in order to maintain good compliance, it is important to address fear in the community about a link between EVD and MDA as seen in Liberia [13] and apply simple hygiene practices such as not sharing cups of water when taking drugs during MDA. This should be reinforced through the MRU mechanisms.

### General lessons learned

An analysis of the practical steps taken in this example of cross-border collaboration yields a general set of lessons learned that can be applied to the range of political, administrative, operational, technical and financial elements of disease control programs. While each cross-border scenario is unique and each element may not be relevant in every situation, stakeholders engaged in confronting the need for cross-border collaboration for NTDs and other public health challenges should consider these options as levers that can be applied to strengthen their programs and lead to improved outcomes.

### Political

- Achieve political commitment and authorization for collaboration at the highest level possible/necessary in each affected country
  - ○ Link on-going disease control efforts to higher-level political influence bodies to raise importance on the national agendas

### Administrative

- Establish a guidance document that identifies the common objective of the collaboration and provides an objective reference of identified key issues for all parties to refer to in the course of the collaboration.
  - ○ All written communications should be provided in the official languages of each participating country.

### Operational

- Agree on a regular cycle of national disease task force strategy meetings, at a minimum annually.
- Conduct more frequent cross-border committee meetings between district medical officers and associated partner organizations (e.g., NGDO) program teams to align on tactical implementation
  - ○ Account for these statutory meetings in each country’s budgeting process and work plans
- Share existing training resources and co-develop new resources to ensure sufficient and similar health care worker training
- Align each county’s drug procurement requests to ensure adequate and non-duplicative coverage for border regions, also considering cross-border migration issues

### Technical

- Create a mechanism to provide complementary technical/scientific support as necessary
- Jointly institute strong Monitoring and Evaluation programs to ensure that all endemic villages are treated.
- Develop joint data management and common reporting of treatment coverage and impact on disease.

### Financial

- Advocate for and mobilize resources for the implementation of the common elements of the program.

### Applying the lessons learned

For onchocerciasis in Africa, there are several clear cross-border scenarios that will need to be actively addressed in order for country-level disease control and elimination goals to be achieved. Examples include the border areas between other West African countries, Malawi and Mozambique, Tanzania and Uganda, and Ethiopia and Sudan. For other NTDs, especially the NTDs that are eligible for preventive chemotherapy (PC-NTDs) with stated control and elimination targets, the same considerations and principles apply. These PC NTDs are trachoma, soil transmitted helminths, schistosomiasis, lymphatic filariasis and onchocerciasis. To ensure adequate consideration is given to this important issue, the disease-specific programs that support the PC NTDs can evaluate their program references for inclusion of specific resources to inform and support cross-border collaboration. At the country level, neighboring countries should consider putting in place general cross-border collaboration frameworks based on the principles contained herein that can be activated and customized as necessary. Finally, WHO could augment their existing resolution on cross-border collaboration by providing a framework and guidance that countries and operational partners can implement.

## Conclusions

As long as diseases are not confined to national borders, the need will continue for effective cross-border collaboration to fight those diseases. The lessons learned from the onchocerciasis program in the contiguous areas of the countries of the Mano River Union provide a framework of principles that can be applied to other cross-border situations, not just for onchocerciasis but also for other PC NTDs. The principles cover a range of considerations: Political, administrative, operational, technical, and financial. All partners and stakeholders involved in these programs—WHO, national NTD programs, implementation NGDOs—should actively consider how they can adapt and apply these principles in each unique geographic and political scenario. The importance of clearly applying these principles will increase as the global NTD community focuses on the targets reflected in the WHO Roadmap on NTDs and the London Declaration. Successful efforts to address cross-border challenges will enhance the collective response to control and eliminate onchocerciasis and other NTDs, resulting in improved health for the more than one billion people affected [5].

## Abbreviations

CDD, community directed distributor; MDA, mass drug administration; NGDO, non-governmental development organization; NTD, neglected tropical diseases; PC, preventive chemotherapy; WHO AFRO, African regional office of the World Health Organization; WHO, World Health Organization

## Footnotes

1 Partners supporting NTD implementation in the 4 countries include: In Sierra Leone: United States Agency for International Development (USAID), END in Africa, FHI360, Helen Keller International (HKI), the Centre for Neglected Tropical Diseases (CNTD) and Sightsavers; In Liberia: CNTD, Sightsavers and the Organization for the Prevention of Blindness (OPC); In Guinea: HKI, Filarial Programmes Support Unit (FPSU), Sightsavers and OPC; in Cote d’Ivoire: HKI, FPSU and Sightsavers.
